# Comparative Analysis of Salmon Cell Lines and Zebrafish Primary Cell Cultures Infection with the Fish Pathogen *Piscirickettsia salmonis*

**DOI:** 10.3390/microorganisms9122516

**Published:** 2021-12-06

**Authors:** Javiera Ortiz-Severín, Julia I. Tandberg, Hanne C. Winther-Larsen, Francisco P. Chávez, Verónica Cambiazo

**Affiliations:** 1 Laboratorio de Microbiología de Sistemas, Departamento de Biología, Facultad de Ciencias, Universidad de Chile, Santiago 7800003, Chile; javiera.ortiz@inta.uchile.cl (J.O.-S.); fpchavez@uchile.cl (F.P.C.); 2 Laboratorio de Bioinformática y Expresión Génica, Instituto de Nutrición y Tecnología de los Alimentos, Universidad de Chile, Santiago 7830489, Chile; 3 Center of Integrative Microbiology and Evolution, University of Oslo, 0316 Oslo, Norway; tandberg98@gmail.com (J.I.T.); h.c.winther-larsen@farmasi.uio.no (H.C.W.-L.); 4 Department of Pharmacology and Pharmaceutical Biosciences, School of Pharmacy, University of Oslo, 0316 Oslo, Norway; 5 Fondap Center for Genome Regulation, Universidad de Chile, Santiago 8370415, Chile

**Keywords:** *P. salmonis* virulence, cell culture viability, host–pathogen interaction, zebrafish, kidney primary cell culture, salmon cell lines, infection biomarkers

## Abstract

*Piscirickettsia salmonis* is the etiologic agent of piscirickettsiosis, a disease that causes significant losses in the salmon farming industry. In order to unveil the pathogenic mechanisms of *P. salmonis*, appropriate molecular and cellular studies in multiple cell lines with different origins need to be conducted. Toward that end, we established a cell viability assay that is suitable for high-throughput analysis using the alamarBlue reagent to follow the distinct stages of the bacterial infection cycle. Changes in host cell viability can be easily detected using either an absorbance- or fluorescence-based plate reader. Our method accurately tracked the infection cycle across two different Atlantic salmon-derived cell lines, with macrophage and epithelial cell properties, and zebrafish primary cell cultures. Analyses were also carried out to quantify intracellular bacterial replication in combination with fluorescence microscopy to visualize *P. salmonis* and cellular structures in fixed cells. In addition, dual gene expression analysis showed that the pro-inflammatory cytokines IL-6, IL-12, and TNFα were upregulated, while the cytokines IL1b and IFNγ were downregulated in the three cell culture types. The expression of the *P. salmonis* metal uptake and heme acquisition genes, together with the toxin and effector genes *ospD3*, *ym*t, *pipB2* and *pepO*, were upregulated at the early and late stages of infection regardless of the cell culture type. On the other hand, Dot/Icm secretion system genes as well as stationary state and nutrient scarcity-related genes were upregulated only at the late stage of *P. salmonis* intracellular infection. We propose that these genes encoding putative *P. salmonis* virulence factors and immune-related proteins could be suitable biomarkers of *P. salmonis* infection. The infection protocol and cell viability assay described here provide a reliable method to compare the molecular and cellular changes induced by *P. salmonis* in other cell lines and has the potential to be used for high-throughput screenings of novel antimicrobials targeting this important fish intracellular pathogen.

## 1. Introduction

*Piscirickettsia salmonis* is a Gram-negative, facultative intracellular bacterium [1,2] that causes salmonid rickettsial septicemia (SRS) or piscirickettsiosis, an infectious disease that affects diverse fish species worldwide and causes significant economic losses in aquaculture [3,4,5]. First described as an epizootic disease, causing high mortality in the southern region of Chile, *P. salmonis* was responsible for a cumulative mortality of up to 70% in coho salmon (*Oncorhynchus kisutch*) in 1989 [6]. Since then, SRS disease has been reported in every species of farmed salmonids (chinook salmon, *Oncorhynchus tshawytscha*; masu salmon, *Oncorhynchus masou*; rainbow trout, *Oncorhynchus mykiss*; Atlantic salmon, *Salmo salar*) [7,8] and other non-salmonid hosts, such as the European sea bass *Dicentrarchus labrax* [9], the white seabass *Atractoscion nobilis* [10], the Hawaiian tilapia [11], and a variety of native wild fish in southern Chile [12], among others. Since its discovery, *P. salmonis* has caused annual outbreaks in the southern Chilean region, being the principal cause of infection-related deaths in the industry [13]. Considering the multitude of hosts that *P. salmonis* can infect, numerous efforts have been made to understand the critical mechanisms involved in the host–pathogen interactions.

Currently, vaccines and antibiotics are the main strategies for prevention and treatment against SRS. Still, the limited effectiveness of current management highlights the need to develop novel approaches to prevent this disease and combat this intracellular pathogen. In addition, *P. salmonis* infections are the main cause of the use of over 300 tons of antibiotics per year in the Chilean salmon farming industry [14], which corresponds to 100 times the amount of antibiotics used in the Norwegian salmon farming industry [15], generating an ecological impact in the region. Furthermore, this strategy is associated with increased bacterial antibiotic resistance and a resurgence of opportunistic infectious diseases in fish [16,17]. Consequently, innovative therapeutic strategies against SRS are highly desired. Considering this, the establishment of robust models that resemble the *P. salmonis* infection cycle in laboratory conditions to study the bacterial infection process, and the host response to *P. salmonis* infection, will be highly advantageous.

*P. salmonis* is a versatile pathogen that has been proven to infect different cell lines with different origins in vitro. This includes cell lines derived from a variety of tissues from Atlantic salmon, Coho salmon, Chinook salmon, Rainbow trout, common carp, and even insect cells [7,8,18,19,20]. Considering that *P. salmonis* infection in vitro is not restricted exclusively to salmonid cell lines, we aimed to examine whether the zebrafish primary cell culture could be used to study the *P. salmonis* infection cycle. The zebrafish (*Danio rerio*) is a small tropical teleost that has been gaining popularity as a model organism to study bacterial pathogenesis. Until now, *P. salmonis* infection assays have been carried out in adult zebrafish individuals [21] and primary cell cultures from zebrafish tissues to study the proteome of *P. salmonis* membrane vesicles upon infection, and their role in fish immunity [22]. However, studies regarding the bacterial infection process in the infected cells are still lacking. In salmon cell lines, *P. salmonis* infection is characterized by the production of *Piscirickettsia*-containing vacuoles (PCV), the intracellular compartment where the bacteria replicates, and concludes with the cell lysis [23]. Even though this strong cytopathic effect has been described, no reliable infection assay for high-throughput virulence screening is available for this critical fish pathogen [8,23,24]. Thus, in this work, we implemented a microplate-based assay that uses a cell viability reagent (alamarBlue) to track the cell status upon *P. salmonis* infection.

The present study aims to establish a robust cell model that resembles the *P. salmonis* infection cycle in laboratory conditions in order to study the bacterial infection process together with the host response to *P. salmonis* infection. With that purpose, we compared host viability changes during *P. salmonis* infection in two different Atlantic salmon-derived cell lines, with macrophage and epithelial cell properties, and zebrafish primary cell cultures, using the alamarBlue reagent. Intracellular bacterial replication was quantified by quantitative PCR (qPCR) and visualized by fluorescence microscopy analyses of cellular structures in fixed cells. In addition, dual gene expression analysis of infection biomarkers was used to follow the expression of known *P. salmonis* virulence factors and host immune-related genes. Our investigation gives the first insights into high-throughput in vitro virulence assays against SRS and opens the door for the future development of novel molecules targeting *P. salmonis*.

## 2. Materials and Methods

### 2.1. Bacteria, Media, and Growth Conditions

*P. salmonis* LF-89 (type-strain ATCC VR 1361) was obtained from the American Type Culture Collection (ATCC) and routinely grown at 18 °C on Austral-SRS broth [25] with agitation. For infection experiments, bacteria were recovered from Austral-SRS agar plates and used to inoculate Austral-SRS broths. Cultures were incubated in a shaking incubator at 140 rpm and 18 °C for 3 days (exponentially growing bacteria) or 7 days (stationary-state bacteria) before harvesting for infection experiments and RNA purification (Figure S2).

### 2.2. Infection and Culture Conditions for Salmon-Derived Cell Lines

Two cell lines derived from Atlantic salmon kidney were used in this study, Salmon Head Kidney-1 (SHK-1) macrophage-like cells (General Cell Collection n° 97111106) and Anterior Salmon Kidney (ASK) epithelial-type cells (ATCC CRL-2747™). Both cell lines were cultured at 20 °C in Leibovitz’s L-15 medium (Gibco, Thermo Fisher Scientific, Waltham, MA, USA) supplemented with 10% fetal bovine serum (FBS, Hyclone, Thermo Fisher Scientific, Waltham, MA, USA), 2 mM L-glutamine (Gibco), and 40 µM β-mercaptoethanol. For infection assays, SHK-1 cells (passage 48 to 52) and ASK cells (passage 73 to 77) were seeded at 80–90% confluency and attached overnight at 20 °C. The culture media was replaced with fresh L-15 with 5% FBS before infection with stationary-state bacteria at a multiplicity of infection (MOI) of 100:1 bacteria/cell ratio, or mock-infection with sterile Austral-SRS medium, following a previously reported infection protocol [23]. After three days of co-incubation at 16 °C, gentamicin was added 1 h to 50 µg/mL final concentration to kill extracellular bacteria. The infection assays (in triplicate) were monitored daily under an inverted optical microscope (100× magnification) for up to 20 days. The growth and infection temperature used for the cell lines was set at 20 and 16 °C, respectively, as previously reported [26].

### 2.3. Zebrafish Husbandry and Generation of Zebrafish Primary Cell Cultures

Zebrafish wild-type strain AB, male and female, were obtained from the model fish unit at the Aleström Zebrafish Lab in the Norwegian University of Life Science. The fish were fed every morning with brine shrimp (Scanbur AS, Nittedal, Norway) and were fed SDS 400 Scientific Fish Food (Scanbur AS, Nittedal, Norway) in the afternoon. The water was provided by the model fish unit at the Norwegian University of Life Science and was supplemented with 0.55 g/L Instant Ocean Sea salt, 0.053 g/L Sodium Bicarbonate, and 0.015 g/L Calcium Chloride. Water parameters were monitored every third day using commercial test kits (TetraTest kit): pH, NO^2−^, NO_3_^2^, NH_3_/NH_4_^+^ and water hardness. The tanks were housed in a water system with a controlled temperature (28 °C) and a cycle consisting of 14 h of light and 10 h of darkness. Zebrafish experiment was approved by NARA (The Norwegian Animal Research Authority) and the wastewater was decontaminated by chlorination and tested for sterility before disposal. For the experiments, adult fish were sacrificed by an overdose of tricaine methanesulfonate (250 mg/mL) before whole kidney marrow and spleen were isolated as described [27]. Groups of ten organs (kidneys or spleens) were immediately submerged in L-15 with 5% FBS supplemented with PenStrep (Penicillin-Streptomycin 5.000 U/mL, Thermo Fisher Scientific). The tissue was mechanically disaggregated with a sterile 40 µm Nylon cell strainer (Corning, Sigma-Aldrich, Darmstadt, Germany), homogenized by pipetting, and individual cells were enumerated by trypan blue exclusion using a Bürker chamber. Homogenized cells from ten kidneys or spleens were seeded in T25 culture flasks or 96-well plates and incubated 24 h at 20 °C with fresh L-15 with 5% FBS supplemented with PenStrep and gentamicin (50 µg/mL). After 24 h, the culture’s supernatant was collected for cell counting, and the number of attached cells was calculated by subtracting the loose cells in the supernatant from the seeded cells. All of the subsequent assays were performed at 20 °C, as reported previously [22].

### 2.4. Standardization of Cell Viability Assays

Cell viability was quantified in infected and uninfected cell cultures using the alamarBlue^®^ non-toxic colorimetric indicator (alamarBlue Cell Viability Reagent, Thermo Fisher Scientific). alamarBlue was designed to quantify the cell proliferation [28] and toxicity of agents [29] in mammalian cells, so the protocol was adjusted for the use of fish cells in infection assays with intracellular bacteria, as follows. Fluorescence measurements were used as they require fewer calculations and are more sensitive due to considerable overlap of the absorbance spectra for oxidized and reduced dye forms. Calibration curves were used to monitor the alamarBlue reduction by different number of cells by measuring fluorescence at 530–550 nm for excitation and 600 nm for emission in a CLARIOstar microplate reader (BMG LABTECH, Ortenberg, Germany). Two-fold dilutions of cells were seeded in 96-well white plates in L-15 media supplemented with 10% FBS and a mixture of antibiotics (Pen-Strep + gentamicin) in the zebrafish primary cell cultures. After cellular attachment (18–24 h at 20 °C), the medium was replaced with the alamarBlue solution in L-15 media with 5% FBS. Incubation was carried on for 4, 6, 8, 24 and 48 h before fluorescence quantifications, at 16 °C for the SHK-1 and ASK cells, and 20 °C for the zebrafish primary cell cultures. Fluorescence (in arbitrary units) was measured for different numbers of seeded cells, and the average of six replicates, with their corresponding standard deviations, was used.

### 2.5. Viability Assays in Infected Cell Cultures

As described above, infection assays were performed for SHK-1 and ASK cells in white 96-well plates (Nunc™, Thermo Fisher Scientific). For SHK-1 and ASK cell lines, 1 × 10^4^ cells/well were seeded in L-15 with 5% FBS in six wells per experiment, with four biological replicates. After the attachment of the cells, cultures were incubated with *P. salmonis* (MOI = 100) or sterile Austral-SRS for three days at 16 °C. Afterward, the cultures were washed, and the extracellular bacteria were removed by gentamicin treatment (day 0). The culture’s viability was monitored over time and quantified every four days with the alamarBlue method as mentioned above.

Infections in the zebrafish kidney primary cell cultures (ZKPCC) were conducted in white 96-well plates with 3 × 10^5^ seeded cells/well. After 24 h of incubation with antibiotics for cell attachment, cultures were washed to remove antibiotics and to estimate the number of attached cells as described above. For infection assays, five wells per experiment with three biological replicates were used. Cultures were incubated at 20 °C for 24 h with stationary-state bacteria (*P. salmonis* MOI = 100) or sterile Austral-SRS, followed by gentamicin treatment. The primary cell culture’s viability was monitored over time and quantified every two days using the same parameters as the calibration curve for alamarBlue reduction. Irrespective of the cell culture type, at least six replicates were used for each independent experiment (N = 3), and the viability was plotted as a percent of the fluorescence emitted by the cultures at the beginning of the assay (day 0), following the formula:

(1) $$\mathrm{Percent}\;\mathrm{viability}\;\left(\%\;\mathrm{reduced}\;\mathrm{alamarBlue}\right)=\left(\frac{\mathrm{Fluorescence}\;\left(\mathrm{AU}\right){\;\mathrm{day}}_{i}}{\mathrm{Fluorescence}\;\left(\mathrm{AU}\right){\;\mathrm{day}}_{0}}\right)\times 100$$

where *i* = 3, 6, 9, 12 or 15 days for cell lines and 2, 4, 6, 8 or 12 days for ZKPCC. Day 0 refers to the moment after gentamicin treatment.

### 2.6. Immunofluorescence Microscopy and Antibody Staining

SHK-1, ASK and ZKPCC were seeded in 8-well chambers for microscopy visualization (Lab-TekII Chamber slide with cover RS Glass slide sterile, Thermo Fisher Scientific), using 4 × 10^4^ cells/well for the cell lines, and 2 × 10^5^ cells/well for ZKPCC. The culture and infection conditions were the same as described above. Infected and mock-infected cell cultures were fixed with 4% paraformaldehyde for 10 min at 6- and 12-days post-infection (dpi) for the cell lines and 1- and 5-dpi for ZKPCC. After fixation, cultures were washed with PBS, permeabilized with 0.5% Triton X-100 in PBS for 10 min and incubated for 2 h with blocking solution (5% goat serum in PBS). *P. salmonis* was detected by incubation with specific anti-*P. salmonis* antibodies (SRS-Fluorotest indirect, BiosChile S.A, Santiago, Chile), diluted in blocking solution 1:50 for cell lines and 1:200 for ZKPCC. Incubation was carried out overnight with mild agitation and protected from light. Afterward, the primary antibody was removed, and cultures were washed three times with PBS before incubating with the secondary antibody (goat anti-rabbit coupled with AlexaFluor 594^®^), diluted 1:500 for cell lines and 1:1000 for ZKPCC. The secondary antibody was incubated for 2 h protected from light and washed three times with PBS before actin-staining for 30 min (100 nM phalloidin: AlexaFluor 488^®^ in blocking solution). Cell nuclei were stained with DAPI and mounted with ProLong™ Gold Antifade Mountant with DAPI (Life Technologies). Mounted cells were kept at 4 °C in the dark, images were acquired in an Olympus FV1000 Confocal microscope using FV10-ASW Viewer software, and Adobe Illustrator version 25.3.1 was used to diagram the images and compose the figures.

### 2.7. RNA Extraction from P. salmonis and Cell Cultures

*S. salar*, *D. rerio* and *P. salmonis* total RNA were purified from infected and non-infected fish cells, and intracellular and broth-cultured bacteria. Infections were carried out as mentioned above in T25 flasks for SHK-1 and ASK cell lines after 6- and 12-dpi, and for ZKPCC after 1- and 5-dpi. Three biological replicates were used for each infected and mock-infected cell culture, and for *P. salmonis* broth cultures, using 1.5 mL of three-day old cultures in Austral-SRS medium. Bacterial cells were pelleted by centrifugation at 8000× *g* for 5 min in a Hettich Rotina 420R (v01.11 swing out rotor 4723) and washed with PBS before suspension in RLT buffer (RNeasy^®^ Mini Kit, Qiagen). Bacteria were disaggregated and homogenized with a 27 gauge syringe and RNA extraction was conducted following the manufacturer’s instructions. For infected and mock-infected cell cultures, cells were collected by trypsinization of cultures followed by centrifugation at 4000× *g* for 5 min. Bacteria were recovered from cell cultures by collecting the supernatant removed before trypsin treatment and centrifuging at 8000× *g* for 5 min. Culture supernatant and cell pellets were pooled together before adding RTL buffer. Cells with and without bacteria were homogenized with a 27gauge syringe and RNA extraction was conducted following the manufacturer’s instructions.

RNA was eluted in nuclease-free water and incubated for 30 min at 37 °C with RNase-Free DNase I (Ambion) to remove residual gDNA. The quantity and quality of RNA were determined by measuring absorbance in a Picodrop V2.07 microliter UV/VIS spectrophotometer. All samples used had a 260/280 ratio between 1.98 and 2.43 (Table S1). The same amount of RNA (470 ng) from the *P. salmonis* cultures, SHK-1, ASK and ZKPCC (infected and mock-infected cultures) were used as starting materials to synthesize cDNA, using the High-Capacity RNA-to-cDNA kit (Applied Biosystems).

### 2.8. Quantitative Real-Time PCR Assays (qPCR)

Primers for *P. salmonis* transcripts were described in a previous work conducted by our group [26]. *S. salar* specific primers for immune-related genes, or for genes with altered expression during *P. salmonis* infection were previously reported in the references included in Table S2. These primers were synthetized by Macrogen (South Korea). To quantify most of the *D. rerio* immune-related genes, QuantiTec bioinformatically validated primers were obtained from Qiagen (Hilden, Germany); the remaining primers were obtained from Life Technologies Inc. (Carlsbad, CA, USA). The complete list of *P. salmonis*, *S. salar* and *D. rerio* primers is included in Table S2. 

For qPCR assays, cDNAs were diluted to 100 ng. *S. salar* and *D. rerio* transcripts in *P. salmonis*-infected and mock-infected conditions were quantified in a LightCycler^®^ 480 (Roche) using the Power™ SYBR™Green Master Mix kit (Applied Biosystems, Thermo Fisher Scientific, Waltham, MA, USA). Bacterial transcripts (in pure bacteriological cultures or in infected cell cultures) were quantified in an AriaMx Real-Time PCR System (Agilent) using the Takyon™ qPCR Master-Mix kit (Eurogentec) and the Agilent AriaMx 1.0 software. PCR conditions were 95 °C for 3 min followed by 95 °C for 3 s, 60 °C for 15 s, and 72 °C for 15 s for a total of 40 cycles. Melting curves (1 °C steps between 60 and 95 °C) ensured that a single product was amplified in each reaction. The geometric median of the housekeeping genes was calculated for each sample, and used to calculate the relative expression levels of the genes using the method described by Pfaffl [30], with two housekeeping genes being used as an internal reference (*recF* and *rho* for *P. salmonis*, and *18S* and *eef1a* for *S. salar* and *D. rerio*). Three biological replicates were analyzed, and PCR efficiencies were determined by linear regression analysis performed directly on the sample data using LinRegPCR [31]. GraphPad Prism software version 6.01 was used for graphical representation of the results.

### 2.9. P. salmonis Quantification inside Cell Cultures

Intracellular bacteria were quantified by qPCR of infected SHK-1, ASK and ZKPC cultures after total RNA purification, using a standard curve. The expression of the bacterial housekeeping genes *recF* and *rho* was used to construct the curve by extracting total bacterial RNA from different numbers of bacterial cells. For this, bacteria from an exponentially growing culture in Austral-SRS medium were quantified using a Petroff-Hausser chamber, bacteria were diluted, and RNA was purified and quantified using the Takyon™ qPCR Master-Mix kit (Eurogentec) in an AriaMx Real-Time PCR System (Agilent) as described above. The standard curve was constructed with the number of bacterial cells and the Ct value of the *recF* and *rho* genes. Ct values of the intracellular bacteria from infected cultures were interpolated in the curve to obtain the bacterial burden inside the different hosts.

### 2.10. Statistical Analysis

GraphPad Prism software version 8.0.1 for Windows (GraphPad Software, La Jolla, CA, USA, www.graphpad.com) was used for graphical representation and statistical analysis of the results. Differences between groups were compared by the unpaired two-tailed Student’s *t*-test. Survival rate was compared using 2-way ANOVA with Sidak’s correction for multiple comparisons, whereas for relative gene expression, a 2-way ANOVA with Fisher’s correction for multiple comparisons was used. *p*-values < 0.05 were considered as statistically significant. Data were expressed as mean ± SD from independent replicates.

## 3. Results and Discussions

To expand upon the existing toolset available to the *P. salmonis* research community, we developed a high-throughput screening method to study *P. salmonis* infection in different hosts using a cell viability reagent, including salmon-derived cell lines and primary cell culture systems of an alternative animal model, the zebrafish. In order to establish zebrafish as a suitable model to study *P. salmonis* infection, we sought to compare the infection generated by the bacterium in the primary cell cultures and the well-studied *S. salar* cell lines, SHK-1 and ASK. For this purpose, the viability, phenotype and expression patterns of marker genes were evaluated in infected and uninfected cultures.

### 3.1. Viability of Cell Cultures over Time

The viability of cell cultures was monitored using the alamarBlue reagent, a blue weakly fluorescent indicator dye that changes to highly fluorescent pink in response to irreversible chemical reduction as a result of the metabolic activity of the cell. Although the fluorescence and the absorbance can be quantified with this method, it is recommended to use fluorescence as it requires fewer calculations and is more sensitive due to considerable overlap of the absorbance spectra for oxidized and reduced forms of dye [28]. Due to its low toxicity, this method has been used to estimate cell viability and proliferation in different cell cultures, including mammalian cell lines and a primary cell culture from rainbow trout fish [29,32,33,34].

**Figure 1:**
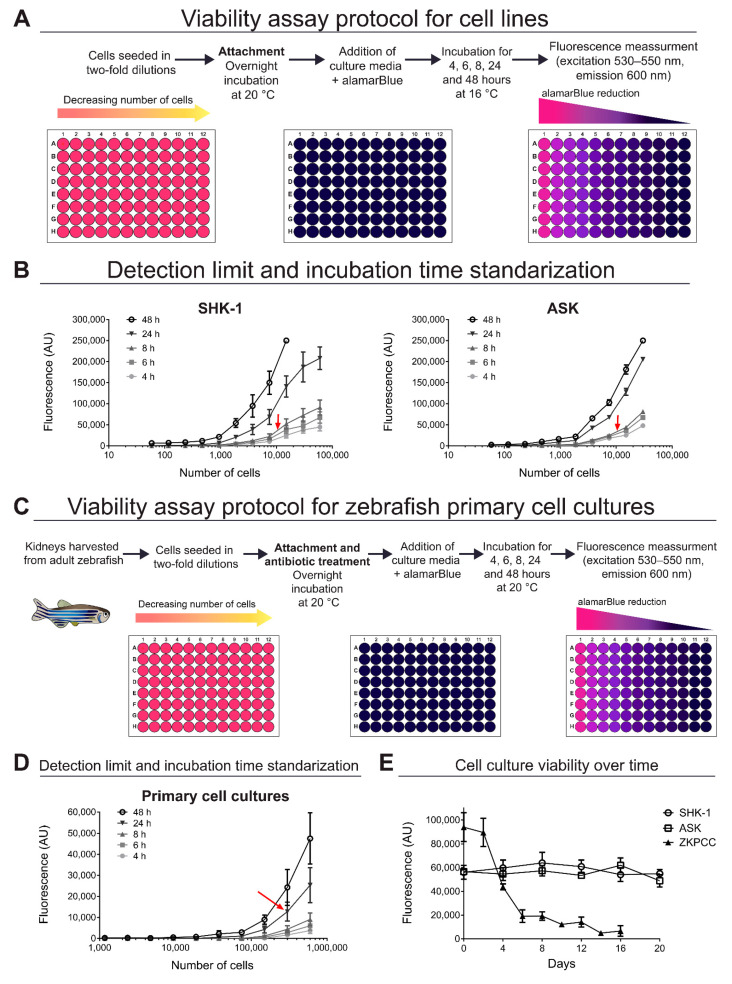
Standardization of cellular viability assays. Schematic representation of the protocol to monitor cell viability with alamarBlue in SHK-1 and ASK salmon cell lines (**A**), and for zebrafish kidney primary cell cultures (ZPCC) (**C**). Incubation with alamarBlue was carried out for 4, 6, 8, 24 and 48 h before fluorescence quantifications, at 16 °C for the cell lines and 20 °C for the ZKPCC. Fluorescence (in arbitrary units) emitted by different number of seeded cells is shown for the cell lines in (**B**) and ZKPCC in (**D**). Fluorescence was measured at 530–550 nm for excitation and 600 nm for emission. Red arrows indicate the number of cells and the incubation time chosen for subsequent experiments and correspond to 10,000 cells and 8 h of alamarBlue incubation for cell the lines, and 300,000 cells with 24 h of incubation for the zebrafish cultures (ZKPCC). The detection limit corresponds to the last fluorescence measurement different than zero; in the selected conditions, the detection limit was ~150 cells for SHK-1, ~250 cells for ASK and ~30,000 for the kidney primary culture cells. Viability of cells over time, measured as fluorescence emitted after reduction of alamarBlue in the selected conditions for each cell type, is shown for the three types of cultures in (**E**).

To monitor the alamarBlue dye reduction by the cells, fluorescence was measured for different numbers of cells and incubation periods. The modified procedure adapted to fish cell cultures is summarized in Figure 1. Fluorescence was successfully detected in all cell types, and the intensity was dependent on the number of cells and the incubation time (Figure 1B,D). In SHK-1 cultures, the fluorescence was above the technique’s detection limit when incubated for 48 h with 15,000 cells or more. For the SHK-1 and ASK cultures, the linear range in the viability assay for 8 h of incubation was between 15,000 and less than 500 cells. Considering this, the infections assay protocol was set at 10,000 seeded cells incubated for 8 h with alamarBlue.

For the zebrafish primary cell cultures, fluorescence was consistently detected in a broader range of cells only for the kidney-derived cultures (Figure S1). For this reason, only the zebrafish kidney primary cell cultures (ZKPCC) and not the spleen-derived cultures were used in further experiments. For the kidney primary cell cultures, 8 h incubations with alamarBlue were insufficient to detect the fluorescent signal when the number of cells was suitable for the infection assays. Thus, the final protocol was set at 300,000 seeded cells and 24 h of incubation time with the alamarBlue reagent. Between 30,000 and 300,000 seeded cells were detected using this incubation time.

In contrast to the SHK-1 and ASK cell lines, ZKPCC do not propagate in culture conditions [35]. Thus, after establishing the assay conditions, the viability of the seeded cells was monitored. For the ZKPCC, the number of seeded cells was 30-times superior to that of the cell lines due to the difference in the reduction of the reagent by the cell types (Figure 1B,D). As expected, the SHK-1 and ASK viability remained relatively constant over a 20-day period, while the viability of the primary cell cultures decreased rapidly over time (Figure 1E). After seeding the ZKPCC, a significant decrease in cell viability occurred from day 0 to day 6, but afterward, the culture viability remained constant until day 12. This period (0–12 days) was used for the infection assays. In addition, a greater variability between replicates was observed in the ZKPCC viability in the first two days after seeding, but then it stabilized in further days (Figure 1E).

### 3.2. Phenotypic Effects of P. salmonis Infection in Cell Cultures

**Figure 2:**
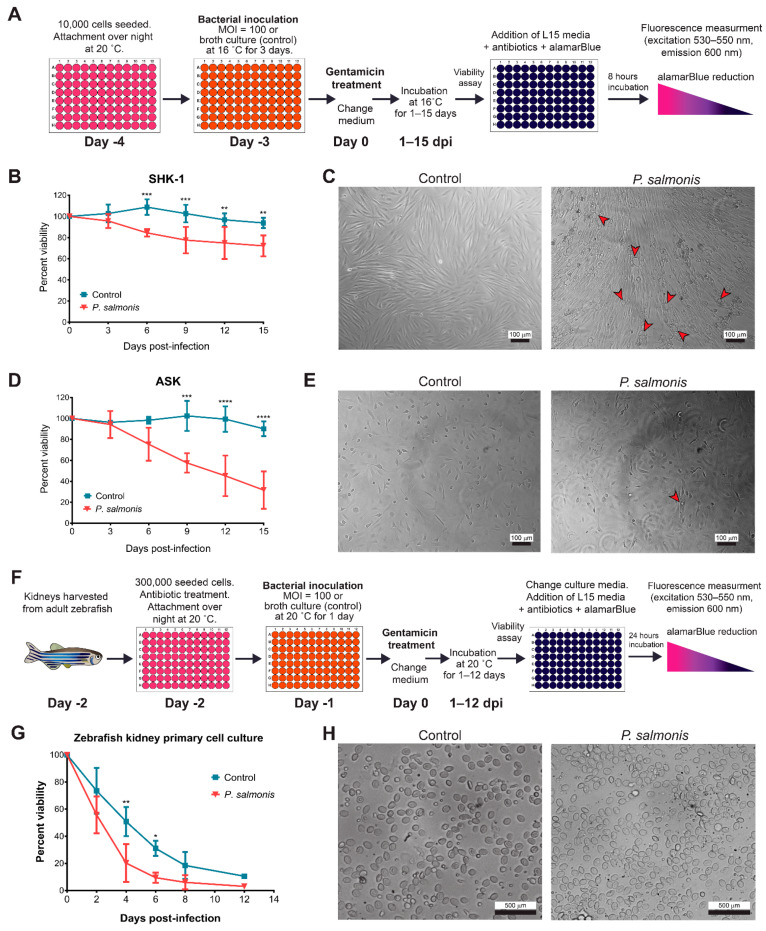
Effect of *P. salmonis* infection in the viability of SHK-1, ASK and zebrafish primary cell cultures. (**A**) Viability assay and infection protocol for SHK-1 and ASK cell lines. After a three-day incubation period with the bacteria, cultures were washed with fresh media supplemented with gentamicin to kill extracellular bacteria, and the cell viability was monitored over time. SHK-1 (**B**) or ASK (**D**) viability over time measured by alamarBlue, and percent viability (the measured viability of a culture at a given time point relative to the viability of cells at day 0 after gentamicin treatment) is shown in the graphs. An average of three independent assays with six wells each, with the correspondent standard deviation, is shown. Asterisks represent statistically significant differences between control and infected cultures at each time point (** *p* < 0.01, *** *p* < 0.001, **** *p* < 0.0001). Representative microphotographs of SHK-1 (**C**) or ASK (**E**) cultures 12 dpi with *P. salmonis* or bacterial culture media as control. Red arrowheads indicate examples of vacuoles. Image with 100× amplification, bar represents 100 µm. (**F**) Viability assay and infection protocol for ZKPCC. After 1 day of incubation with the bacteria, the cells were washed with fresh media supplemented with gentamicin to kill extracellular bacteria and the cell viability was monitored over time. (**G**) ZKPCC cell viability over time measured by alamarBlue. Percent viability represents the measured viability of a culture at a given time point relative to the viability of cells at day 0. The average of three independent assays with five wells each, with the correspondent standard deviation, is shown. Asterisks represent statistically significant differences between control and infected cultures at each time point (* *p* < 0.05, ** *p* < 0.01). (**H**) Representative microphotographs of ZKPCC at 5 dpi to *P. salmonis* or bacterial culture media as a control. Image with 400× amplification, bar represents 500 µm.

To quantitatively compare the infection of *P. salmonis* in different types of cell cultures, we developed a viability assay for infected and mock-infected fish cells (Figure 2A) using the conditions described in Figure 1.

The viability of the SHK-1 cultures infected with *P. salmonis* decreased moderately over time. Still, significant differences were observed after 6 days post-infection (dpi) in comparison to mock-infected cells, reaching less than 80% of the viability of the control cells at 20 dpi (Figure 2B). Viability in *P. salmonis*-infected ASK cultures also decreased significantly compared to mock-infected cells. Unlike the observations with SHK cells, the decrease in viability was more pronounced in the ASK cells and reached less than 30% of the control’s viability at 20 dpi (Figure 2D). The cytopathic effect of the bacterial infection was observed at the culture population level using brightfield microscopy and is shown in Figure 2C for SHK-1 and in Figure 2E for ASK cells. Vacuoles spread throughout the culture were observed in the infected SHK-1 cells (Figure 2C red arrowheads). In the ASK cells, few vacuoles were sporadically detected in infected cultures (Figure 2E red arrowhead). In both cultures, disruption of the cellular monolayer and floating cells was observed at later times (>12 dpi), which corresponds to phenotypes described by other authors [26,36,37]. For further experiments, an early- and a late-stage infection time were selected based on the differences in viability between the *P. salmonis*-infected and the mock-infected cultures, corresponding to 6- and 12-dpi, respectively.

The infection protocol for the zebrafish primary cell cultures is shown in Figure 2F. For the ZKPCC infections, some considerations regarding the origin of the cells had to be taken to establish the infection protocol. Since the zebrafish is a tropical fish that is routinely maintained at 28 °C, the temperature used for infection assays in the SHK-1 and ASK cell lines (16 °C) could not be used, so a higher temperature (20 °C) was used for *P. salmonis* infections in the ZKPCC. At this temperature, bacterial growth is not inhibited or decreased when compared to the optimal temperature for *P. salmonis* growth, 18 °C, although *P. salmonis* was not able to grow at 28 °C, the optimum temperature for zebrafish (Figure S2). Besides temperature, the incubation time was also modified from 3 days before gentamicin treatment in the cell lines to 1 day (Figure 2A,F, respectively) with a MOI = 100, as was used in the cell lines’ infection assays. Although the viability of the mock-infected ZKPCC decreased over time, a significant decrease in the viability of the *P. salmonis*-infected cultures was detected (Figure 2G), with differences of 30.6 and 21.6% in viability between the mock-infected and the infected cultures at 4- and 6-dpi, respectively. Considering that the percent viability of infected cultures decreased over 10% at 6 dpi (Figure 2G), 5 dpi was used to obtain a sufficient number of infected cells in further experiments. In bright-field images, diverse cell types were observed in the primary cell culture, as described previously [35,38]. Due to this characteristic and the fact that the cells were loosely attached and did not form a compact monolayer (as was observed in the cell lines), no apparent phenotypic effects were observed upon infection with *P. salmonis* in the ZKPCC (Figure 2H).

**Figure 3:**
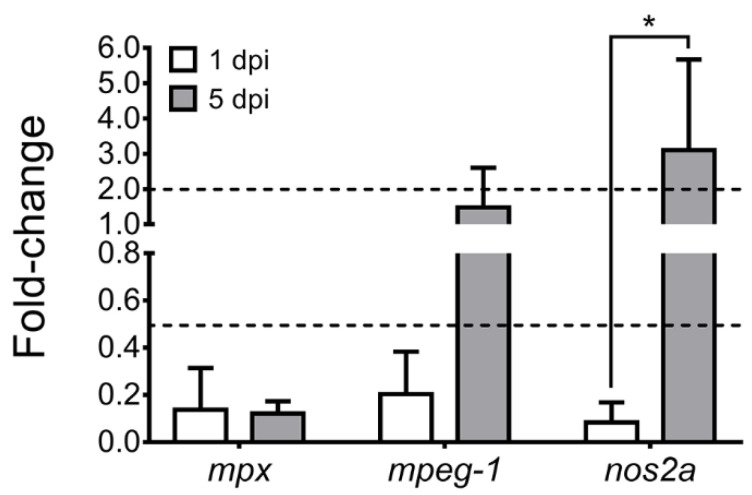
Identification of *mpx*-, *mpeg-1*- and *nos2a*-expressing cells in zebrafish kidney primary cell cultures. Relative gene expression was quantified after 1- and 5-dpi with *P. salmonis* or culture medium as control. Gene expression is expressed as relative to the housekeeping genes. Dotted lines indicate non-significant changes in expression levels (between 0.5- and 2-fold). Asterisks show significant differences compared to control samples (two-way ANOVA with Fisher’s post-test for multiple comparisons, * *p* < 0.05).

Since the ZKPCC is composed of different cell types, we aimed to identify the effect of *P. salmonis* infection in immune-related cells present in the primary cell culture [35,38]. For this, we quantified the expression levels of the zebrafish myeloperoxidase gene (*mpx*) and macrophage-expressed gene-1 (*mpeg-1*), which are marker genes for neutrophils and macrophages, respectively [39]. Additionally, the inducible nitric oxide synthase 2a gene (*nos2a*) was used as a marker for hematopoietic stem and progenitor cells (HSPCs) and the neutrophil- fated progenitor expansion that occurs following infection [39]. Relative gene expression was quantified in *P. salmonis*-infected and mock-infected ZKPCC at 1 dpi (early-stage infection) and 5 dpi (late-stage infection), and a significant decrease in *mpx*, *mpeg-1* and *nos2a* expression was observed at 1 dpi. In contrast, at 5 dpi, only *mpx* decreased significantly, *mpeg-1* did not change its expression and *nos2a* increased (Figure 3). In addition, the expression of *nos2a* rose substantially between the infected and control cells (over 3-fold increase), and between 1- and 5-dpi in *P. salmonis*-infected cells (36.9-fold increase). Thus, *P. salmonis* infection significantly decreased the *mpx*-expressing population of cells at the early and late stages of the infection. The *mpeg-1*-expressing population of cells was also reduced at the early infection stage, but it recovered later at 5 dpi. The *nos2a* gene was induced at the late infection stage in ZKPCC, suggesting the activation of HSPCs and neutrophil expansion in response to *P. salmonis* infection during late-stage infections.

**Figure 4:**
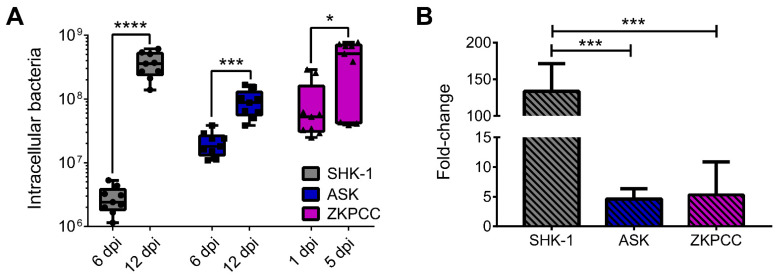
*P. salmonis* quantification inside infected cell cultures. The expression of bacterial housekeeping genes *recF* and *rho* in salmon cell lines (SHK-1 and ASK) or primary cell cultures (ZKPCC) at early- and late-stage infections (6 and 12 dpi for cell lines, and 1 and 5 dpi for ZKPCC) was quantified. Total number of bacteria was inferred from a standard curve (number of bacteria vs. Ct). (**A**) Number of intracellular bacteria at early- and late-stage infections in the three cell cultures. An unpaired t-test between early- and late-infection stages was performed for each cell culture, asterisks show statistical differences (* *p* < 0.05, *** *p* < 0.001 and **** *p* < 0.0001). (**B**) Fold-change of intracellular bacteria between early- and late-stage infections in the three cell cultures. A one-way ANOVA with Tukey’s multiple comparisons test was performed, asterisks show statistical differences (*** *p* < 0.001).

Subsequently, to corroborate that *P. salmonis* was replicating inside the cell cultures, bacterial load was measured by quantitative PCR (qPCR). A standard curve constructed with the expression levels of the housekeeping genes of different numbers of bacterial cells was used. Based on this, the bacterial load during early- and late-stage infection was determined in the salmon cell lines and the ZKPCC. As observed in Figure 4A, the bacterial load increased in the three cell types over time, although the increase between the early- and the late-stage infection was significantly higher in the SHK-1 cells (133-fold increase) when compared to the ASK cells (4.6-fold increase) and the ZKPCC (5.3-fold increase), as shown in Figure 4B. At the late-stage infection, bacterial load in the ASK cell line was significantly lower than that in SHK-1 cells (*p* = 0.0057) and ZKPCC (*p* = 0.0003), indicating that *P. salmonis* does not replicate to the same extent in the epithelial-type cells. As the bacterial growth in broth cultures was similar at the incubation temperature used for the cell lines and the ZKPCC (Figure S2), we do not believe that the difference in bacterial load between the cell cultures is due to temperature. Nevertheless, as the bacterial replication occurs intracellularly, the increase in bacterial load inside the cell cultures indicates that the bacteria were actively replicating inside the tested cell cultures (including the zebrafish cells), which could explain the decreased viability observed in the cell cultures. In addition, this points to a correlation between the cell lines viability and the bacterial load. As mentioned, at the late-stage infection, fewer viable cells were found in the ASK cultures than in the SHK-1 cultures (Figure 2B,E). At the same time, the bacterial load increased over 130 times in the SHK-1 cultures, significantly more than in the ASK cultures. The capacity of *P. salmonis* to maintain the viability of the infected host cells in order to increase its infectivity has been suggested previously. It was postulated that *P. salmonis* inhibits apoptosis specifically in salmon macrophages [18] in order to survive and multiply, which constitutes a crucial part of the infection process in Atlantic salmon disease. This link between the cell culture viability and bacterial load was not that evident in the ZKPCC due to the nature of the primary cell culture, as their viability naturally decreased over time. However, the bacterial load at the late-stage infection was similar between the SHK-1 cultures and the ZKPCC (*p* = 0.9985), indicating that *P. salmonis* was able to replicate efficiently inside the zebrafish cells, obtaining similar bacterial loads to the SHK-1 cells but 7 days earlier.

**Figure 5:**
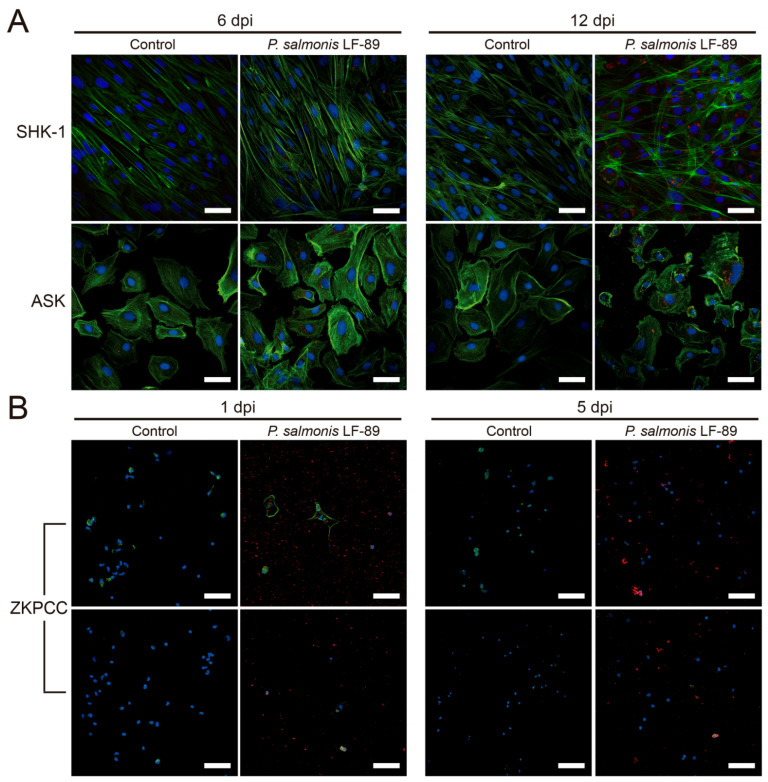
Immunofluorescence microscopy of *P. salmonis*-infected salmon cell lines and zebrafish primary cell cultures. (**A**) *P. salmonis*-infected and mock-infected (control) salmon cell lines SHK-1 and ASK were fixated before fluorescence staining after 6 and 12 dpi. (**B**) *P. salmonis*-infected and mock-infected (control) zebrafish primary cultures (ZKPCC) were fixated after 1 and 5 dpi. Both panels show representative images of zebrafish cells with different cell types. For the three cultures, cellular actin was stained with phalloidin in green (Alexa Fluor 488^®^), and the nucleus in blue with DAPI. Polyclonal antibodies against *P. salmonis* and a secondary antibody coupled with Alexa Fluor 594^®^ marked the bacteria in red. Bar represents 50 µm. Images obtained at each channel are provided in Figure S3.

To visualize the bacteria inside the cells of the three culture types, immunofluorescence microscopy was performed using specific antibodies against *P. salmonis* in SHK-1 and ASK cultures at 6- and 12-dpi (Figure 5A), and in ZKPCC at 1- and 5-dpi (Figure 5B). In all cultures, actin was stained green with phalloidin (Alexa Fluor 488^®^) and the cell nucleus was stained blue with DAPI. The bacteria were successfully detected only in the *P. salmonis*-infected cell cultures. In the salmon-derived cell lines, at 6-dpi, bacterial foci were observed to be spread in the host cells near the actin fibers, followed by the formation of bacterial clusters near the cell nucleus at 12-dpi. This correlates with the formation of replicative vacuoles, or *P. salmonis*-containing vacuoles, where the bacteria replicates inside the SHK-1 cell cultures [23]. Since *P. salmonis* is a small bacterium ranging from 0.5 to 1.5 µm in diameter [1,24], the observed bacterial foci in the cell lines and the ZKPCC most likely correspond to bacterial clusters rather than individual bacteria.

The zebrafish primary kidney cells were smaller than the salmon cells, so the cellular actin was scarce and difficult to observed at the used magnification, and the most prominent cell feature was their nucleus. Despite the use of glass culture chambers treated to improve cell adherence, ZKPCC were loosely attached, and a small percentage of cells remained in the chambers after the fixation and staining protocols. Hence, the low number of cells observed by microscopy does not necessary correspond to an effect of the bacteria. Nevertheless, *P. salmonis* was detected in ZKPCC at 1- and 5-dpi, both outside the cells and in the proximity of the nucleus (Figure 5B). Superposition of the fluorescence signal of cellular structures (nucleus and actin) with the bacteria was also observed in some infected cells (Figure 5B), suggesting the presence of bacterial clusters in the proximity of the cell nucleus, as observed in the infected cell lines. Considering that gentamicin treatment was conducted in all cell types after *P. salmonis* infection, the observed *P. salmonis* cells in the immunofluorescence images had an intracellular origin.

### 3.3. Host Immune Response and P. salmonis Virulence Factors during Infection

The use of transcript levels of selected genes as a biomarker of *P. salmonis* infection in the salmon host was suggested previously [40]. Rise et al. (2004) proposed qPCR-based expression analyses of 19 host genes as molecular biomarkers for *P. salmonis* infection in Atlantic salmon organs (head kidney) and cultured macrophages. Based on a literature search of gene expression analyses (microarray, qPCR or RNA sequencing) of *P. salmonis* infection in salmons [41,42,43] or cell cultures [23,44,45], we selected 15 host targets that were proven to change its expression during *P. salmonis* infection. These targets were used as biomarkers, and their relative gene expression was evaluated in SHK-1, ASK and ZKPCC that was mock-infected and infected with *P. salmonis*. Due to differences between salmon and zebrafish cells in the biomarker’s coding genes, different numbers of genes were tested. For example, type II interferon gamma (*ifnγ* gene in Atlantic salmon) is encoded by ten genes in zebrafish, so the two genes that are most closely related to mammalian interferon gamma (*ifnγ1-1* and *ifnγ1-2*) [46] were evaluated in this host; also, type I interferon was assessed by evaluating the *ifnα* gene in salmon cells and by three *ifnφ* genes (*ifnφ1*, *ifnφ2* and *ifnφ3*) that exert a similar function in zebrafish [47].

**Figure 6:**
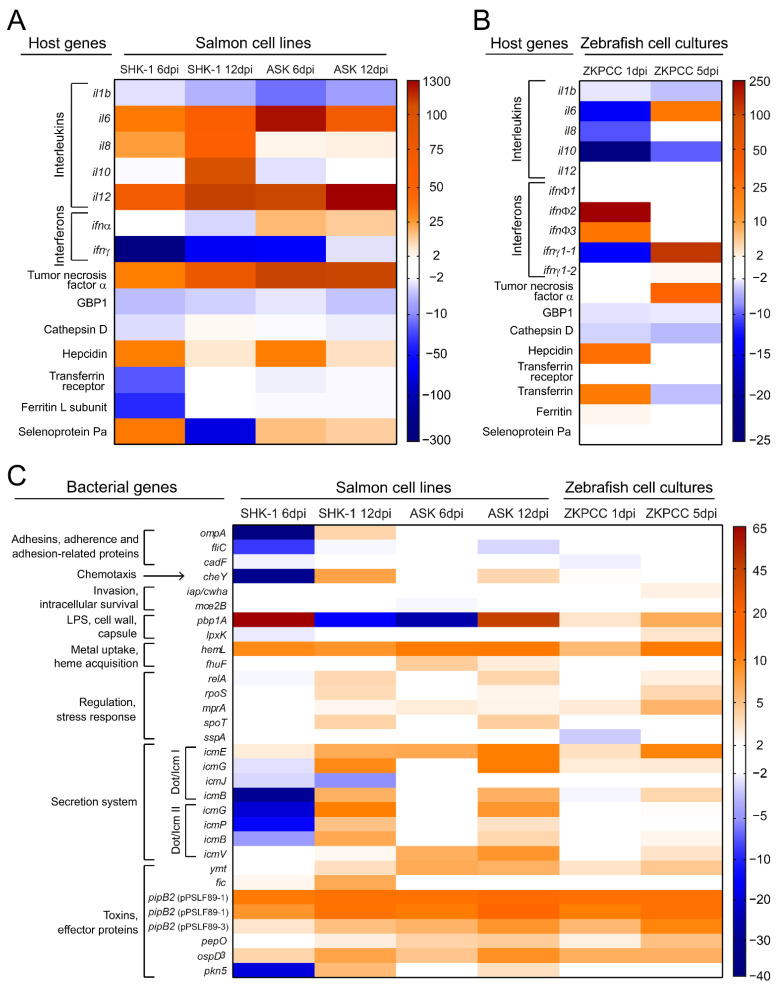
Host and pathogen biomarker’s gene expression during *P. salmonis* infection. Heat map showing relative transcript abundance of immune-related genes in *P. salmonis*-infected cultures and virulence factor genes in *P. salmonis* at early- and late-stage infections. (**A**) Expression level of host biomarkers in SHK-1 and ASK cell lines at 6- and 12-dpi. (**B**) Expression level of host biomarkers in ZKPCC at 1- and 5-dpi. In (**A**,**B**), the gene expression of cell cultures infected with *P. salmonis* is presented as relative to uninfected cell cultures. (**C**) The gene expression of *P. salmonis* biomarkers during infection inside cell cultures is presented as relative to *P. salmonis* growing in Austral-SRS medium. The average of ΔΔCt values for three independent replicates is shown in all heat maps, and non-significant values (between +2- and −2-fold changes) are shown in white.

The immune gene expression profile revealed differential modulation of these markers depending on the host and the infection time, although some genes behaved similarly in salmon and zebrafish cells (Figure 6A,B). In the three cell types, *il1b* transcripts were decreased both in early- and late-stage infection. Similarities were observed between infected SHK-1 and ASK cells as they responded to *P. salmonis* by increasing the expression of genes encoding the pro-inflammatory cytokines IL-6, IL-12 and TNFα at 6 dpi and 12 dpi, whereas in zebrafish cells, expression of *il6* and *tnfα* only increased at the late stage of infection. Thus, tumor necrosis factor alpha (as well as interleukin 1 b and interleukin 6) were proven to be suitable biomarkers of *P. salmonis* infection in the three cell types, as previously suggested [43]. As shown in Figure 6A, SHK-1 displayed an increased *il8* expression at the early stage of infection and remained elevated through 12 dpi, suggesting that this macrophage-like cell line responds to *P. salmonis* by producing neutrophil attractants. The restricted expression of *il8* in SHK-1 cells was consistent with previous reports [43]. In this cell line, *il10* expression also increased in response to *P. salmonis*, which might be a potential anti-inflammatory event promoted by the pathogen to establish a persistent infection. Consistently, previous work in the rainbow trout monocyte/macrophage cell line RTS11 showed high expression levels of *il10* transcripts at different infection times [45].

The interferon alpha biomarker behaved differently in the three cultures, as *ifnα* transcripts decreased in SHK-1 cells at 6 dpi and increased in ASK at both infection stages, whereas in zebrafish, *infφ1* did not change between infected and mock-infected cells, but *infφ2* and *infφ3* increased their expression at 1 dpi. Considering that, in fish, interferon gamma stimulates the expression of cytokines and chemokines associated with the pro-inflammatory response and stimulates the production of nitrogen and oxygen reactive species (NOS and ROS) in phagocytic cells [48], the decreased expression of *ifnγ* in *S. salar* and *ifnγ1-*1 in *D. rerio* suggests a bacterial-mediated mechanism to promote its infection. Previous work demonstrated increased levels of interferon gamma transcripts in head kidney from infected Atlantic salmon [43]. Thus, the increased expression of *ifnγ1-1* in the ZKPCC at 5 dpi reproduces the enhanced interferon gamma expression observed in salmons and validates interferon gamma as a biomarker for *P. salmonis* infection in hematopoietic tissues. In an RTS-11 cell line infected with *P. salmonis*, mRNA levels of GBP1 and cathepsin D transcripts were increased [45], whereas in *S. salar* cells, cathepsin D decreased its expression upon *P. salmonis* infection [23]. Here, we observed a general decreased expression of GBP1 and cathepsin D biomarkers (Figure 6A,B).

Another set of genes evaluated as biomarkers were those related to iron transport (hepcidin, ferritin, transferrin and transferrin receptor), and selenoprotein Pa, which have been consistently detected as differentially expressed in salmon infected with *P. salmonis* [40,42,49]. In SHK-1 cells, the expression of genes encoding transferrin and ferritin decreased only during early-stage infection. In contrast, the expression of these genes in ASK cells was similar to the mock-infected cells (Figure 6A). In zebrafish cultures, only transferrin gene expression changed significantly in infected cells, increasing in early-stage, and decreasing during late-stage infection (Figure 6B). A consistently increased expression of hepcidin at the early stage of infection was detected in SHK-1, ASK and ZKPCC in response to *P. salmonis* infection, followed by a decrease in hepcidin transcripts at the late stage of infection (Figure 6A,B). Hepcidin, the master regulator of iron homeostasis, binds to ferroportin, the only known cellular iron exporter, mediating its internalization and degradation. Thus, upregulation of the hepcidin-encoding gene is expected to conserve intracellular iron stores [50,51], which is beneficial for intracellular pathogens such as *P. salmonis*. Finally, downregulation of the gene encoding selenoprotein Pa was proposed as a viable candidate biomarker for bacterial infection in salmon macrophages [40] and head kidney tissue [42] infected with *P. salmonis*. Here, we observed that this occurred only in SHK-1 cells at late-stage infection, and thus, selenoprotein Pa seems to be a good biomarker only for *S. salar* macrophages, as *selPa* transcripts increased in ASK cells (Figure 6A) and *sepp1a* did not change its expression in the ZKPCC (Figure 6B).

On the other hand, potential *P. salmonis* virulence factors were categorized as shown in Figure 6C, and their expression profiles were tested in the three culture types. For them, a role during intracellular infection in SHK-1 cells was proposed [28], and here, their expression levels were also tested in ASK and ZKPCC (Figure 6C). Adherence- and invasion-related virulence factors, such as *fliC*, *cadF*, *ompA*, *iap/cwha* and *mce2*, were downregulated or did not change between infected and mock-infected cells. In the case of *ompA,* downregulation was only detected in SHK-1 during early-stage infection. Thus, the expression profile correlates with the infection process timeline since we observed that *P. salmonis* was already inside the host cells at the early and late stage of infection in the three cultures.

The *P. salmonis* LPS and peptidoglycan genes showed a different pattern in the cell cultures. The lipid A 4′-kinase gene (*lpxk*) expression was decreased in SHK-1 cells and increased in zebrafish cells, whereas no expression changes were detected inside the ASK cells. In addition, transcript levels of the penicillin-binding protein 1A gene (*pbp1A*) significantly changed in the three cultures but with different dynamics. The role of *pbp1A* as a virulence factor in *P. salmonis* infection is still unknown. However, the significant changes in the gene expression in different cell types suggest that this gene could be used as a biomarker of the intracellular state of *P. salmonis*. In a general view, both clusters of Dot/Icm secretion system genes (shown as Dot/Icm I and Dot/Icm II in Figure 6C) were overexpressed at the late stage of infection in the three cell cultures. In contrast, a marked decrease in gene expression at early stage of infection was observed only in SHK-1 cells, suggesting that the Dot/Icm secretion system is required for infection in the three hosts. Upregulation of Dot/Icm secretion system genes at the late stages of infection in SHK-1 cells was previously reported, and a role of the Dot/Icm components as mediators of the escape of *P. salmonis* from the host cell was suggested [23,52,53]. As observed before [23], stationary state and nutrient scarcity-related genes were found to be overexpressed only in late stage infection in the three cultures. These results validate these genes as biomarkers of late-stage intracellular *P. salmonis* infection. The expression patterns of these genes also validated the temporal differences between the infection periods of salmon and zebrafish cells. Finally, the toxins and effectors *ospD3*, *ymt*, *pipB2* and *pepO* as well as metal uptake and heme acquisition genes significantly increased their expression in the three cell cultures, suggesting a conserved role of these virulence factors that helps in terms of intracellular bacterial survival irrespective of the host cell type. Interestingly, the three plasmidial copies of *pipb2* were overexpressed in SHK-1, ASK and ZKPCC, as observed before with SHK-1 cells [23].

The major findings and comparisons between the genotypic and phenotypic effects of *P. salmonis* infection in the salmon-derived cell cultures (SHK-1 and ASK) and the zebrafish primary culture, together with the bacterial replication and genetic response to the intracellular lifestyle in the three hosts, are summarized in Table 1.

## 4. Conclusions

In this study, we provided a reliable assay to follow changes in host cell viability after *P. salmonis* infection. For the assay, the alamarBlue reagent was used in a 96-well format, which can be easily scaled up for high-throughput screenings of novel antimicrobials targeting this fish pathogen. Cell viability was tested in three different culture types, including two *S. salar* cell lines and a zebrafish primary cell culture, in which *P. salmonis* infection induced the activation of HSPCs and neutrophil expansion. Intracellular growth of *P. salmonis* in the three cell types was recorded and dual gene expression revealed that the pro-inflammatory cytokines IL-6, IL-12, TNFα, IL1b and IFNγ can be suitable biomarkers of host response to *P. salmonis* infection. The expression analysis of putative virulence factors of *P. salmonis* revealed potential biomarkers of the intracellular state of *P. salmonis* (genes encoding toxin and effector proteins) and biomarkers of late-stage intracellular *P. salmonis* infection (Dot/Icm secretion system genes as well as stationary state and nutrient scarcity-related genes).

## Figures and Tables

**Table 1:** Comparative table of *P. salmonis* infection effects in salmon and zebrafish cell cultures.

	SHK-1 Cells	ASK Cells	Zebrafish Kidney Cells
**Actin** * **P. salmonis** * **DNA**	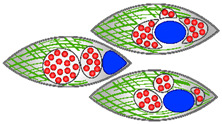	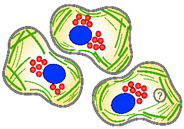	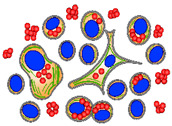
Cell type	*S. salar* head kidney cell line. Phagocytic cells derived from leucocytes, macrophage-like properties.	*S. salar* epithelial cell line derived from kidneys. Epithelial-type cells.	Adult zebrafish kidney primary cell culture. Lymphoid, myeloid, erythroid precursor and stromal cells.
Cellular viability after *P. salmonis* infection	Significant decrease in cellular viability after 6- to 15-dpi with *P. salmonis*. Minimal cell viability of infected cells at 15-dpi, corresponding to 76.9% of control cell viability at 15-dpi.	Significant decrease in cellular viability after 9- to 15-dpi with *P. salmonis*. Minimal cell viability of infected cells at 15-dpi, corresponding to 35.1% of control cell viability at 15-dpi.	Significant decrease in cellular viability after 4- to 6-dpi with *P. salmonis*. Minimal cell viability of infected cells at 6-dpi, corresponding to 30.4% of control cell viability at 6-dpi).
Phenotypic effects of *P. salmonis* infection in cell cultures	Disruption of the cellular monolayer at 12-dpi. Presence of multiple cytoplasmic vacuoles. Different size vacuoles. Actin cytoskeleton alteration. *P. salmonis* clusters in the proximity of cell nucleus and actin fibers.	Disruption of the cellular monolayer at 12-dpi. Presence of few and small cytoplasmic vacuoles. *P. salmonis* presence inside cytoplasmic vacuoles is not confirmed. Actin cytoskeleton alteration. *P. salmonis* clusters in the proximity of cell nucleus and actin fibers.	Cell distribution not disrupted by *P. salmonis* infection. Presence of *P. salmonis* inside and outside the cells. Cytoplasmic vacuoles were not observed. Actin cytoskeleton alteration was not observed. Changes in the number of *mpx* and *mpeg-1* expressing cells. Activation of HSPCs and neutrophil expansion.
Immune response to *P. salmonis* infection	Activation of antimicrobial response: - Increased expression of IL-8. Immune tolerance, anti-inflammatory environment: - Decreased expression of IL-1β, IFN-γ and INF-α - Increased expression of IL-12 and IL-10 (late infection).	Activation of antimicrobial response: - Increased expression of INF-α. Immune tolerance, anti-inflammatory environment: - Decreased expression of IL-1β and IFN-γ- - Increased expression of IL-12. * IL-8 and IL-10 did not change their expression.	Inflammatory environment: - Increased expression of IFN-γ, IL-6, and Nitric oxide synthase 2a (late infection). Immune tolerance, anti-inflammatory environment: - Decreased expression of IL-10 in late infection. * IL-8 did not change its expression.
*P. salmonis* intracellular replication	Intracellular bacteria increased 140-fold in between early and late infection. Bacterial burden: 3.6 × 10^8^ bacteria in late infection.	Intracellular bacteria increased 4.3-fold in between early and late infection. Bacterial burden: 9.2 × 10^7^ bacteria in late infection.	Intracellular bacteria increased 5.3-fold in between early and late infection. Bacterial burden: 4.3 × 10^8^ bacteria in late infection.
*P. salmonis* genes induced during infection	Adherence: decrease in *fliC* at early and late infection, and in *ompA* and *cadF* at late infection. LPS, cell wall, capsule: decrease in *lpxK* and *waaE*, increase in *pbp1A* at early-infection. Metal uptake, heme acquisition: increase in *hemL*. Regulation, stress response: increase in *mprA*, *relA*, *rpoS* and *spoT* at late infection. Secretion systems: decrease at early infection and increase at late infection. Toxin and effectors: *ospD3*, *fic* and *pipB2* increase at early and late infection. *ymt*, *pkn5* and *pepO* increase at late infection.	Adherence: decrease in *fliC* in late infection. Invasion, intracellular survival: decrease in *mce2B* in early infection. LPS, cell wall, capsule: increase in *pbp1A* in late infection. Metal uptake, heme acquisition: increase in *hemL* and *fhuF*-like. Regulation, stress response: increase in *mprA*, *relA*, *rpoS*, *spoT* and *sspA* in late infection. Secretion systems: increase in late infection. Toxin and effectors: *ospD3*, *ymt*, *pipB2* and *pepO* increase in early and late infection, and *pkn5* increase in late infection.	Invasion, intracellular survival: decrease in *cadF* and *mce2B* at early-infection, increase in *iap* in late infection. LPS, cell wall, capsule: increase in *lpxK* in late infection, increase in *pbp1A* in early and late infection. Metal uptake, heme acquisition: increase in *hemL*. Regulation, stress response: increase in *mprA*, *relA* and *rpoS* in late infection. *mprA* increase in early infection, *sspA* decrease in early infection. Secretion systems: increase in late infection. Toxin and effector proteins: *ospD3*, *ymt*, *pipB2* and *pepO* increase in early and late infection.

## Data Availability

Not applicable.

## Supplementary Materials

The following are available online at https://www.mdpi.com/article/10.3390/microorganisms9122516/s1, Figure S1: Reduction of alamarBlue reagent by different numbers of cells present in primary cell cultures isolated from adult zebrafish spleen and kidney tissues, Figure S2: *P. salmonis* LF-89 growth curves in Austral-SRS broth medium at different temperatures, Figure S3: Immunofluorescence microscopy of *P. salmonis*-infected salmon cell lines and zebrafish primary cell cultures, Table S1: Quality of RNA samples measured by absorbance 260/280 ratio, Table S2: Primers used for RT-qPCR in this study.
