# Supplementary material for: Comparative Analysis of Salmon Cell Lines and Zebrafish Primary Cell Cultures Infection with the Fish Pathogen Piscirickettsia salmonis
Source: Microorganisms. 2021 Dec 6;9(12):2516. doi: 10.3390/microorganisms9122516 (PMC8706985; doi:10.3390/microorganisms9122516)
Supplement: Supplementary file 1 [file microorganisms-09-02516-s001.zip › FigureS2.pdf]

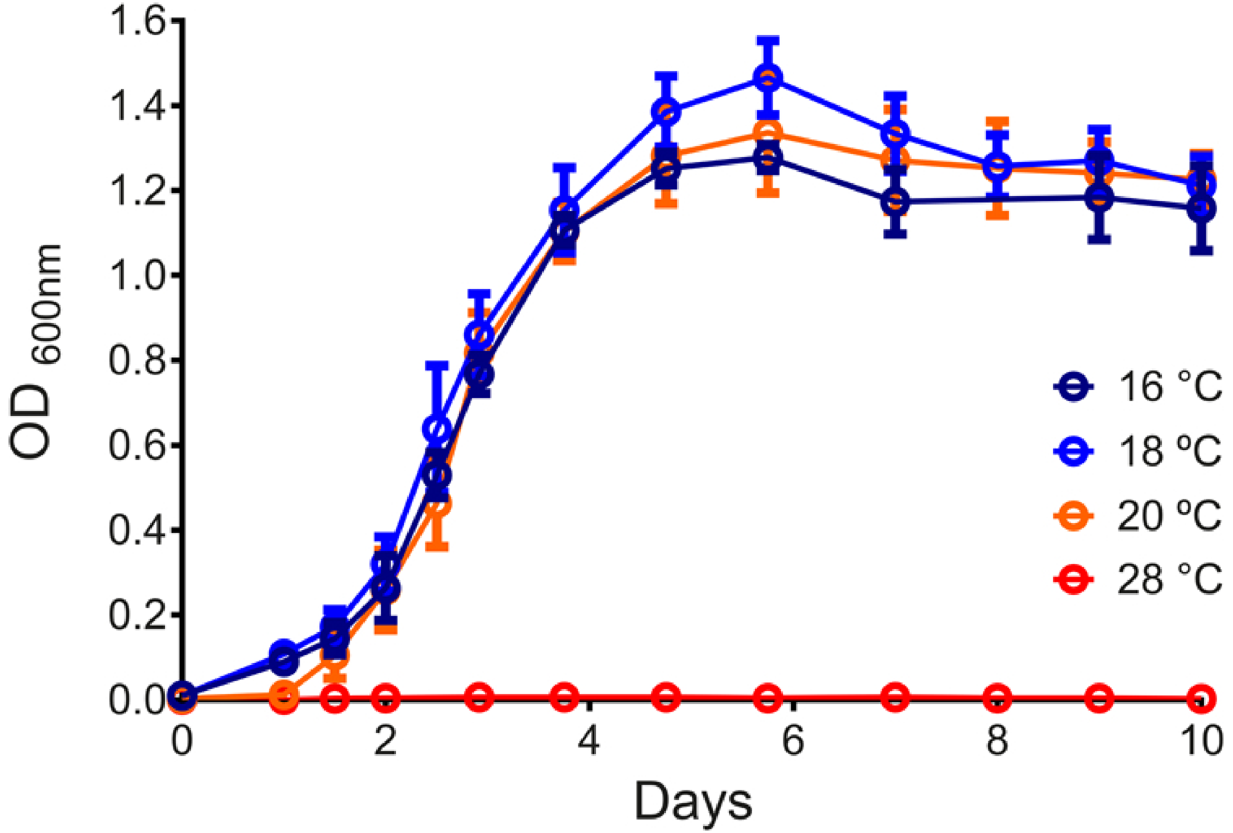


Figure S2. *P. salmonis* LF-89 growth curves in Austral-SRS broth medium at different temperatures. The temperatures used correspond to the cell lines infection temperature (16 ºC), routine bacterial growth temperature (18 ºC), ZKPCC infection temperature (20 ºC), and zebrafish rearing temperature (28 ºC).
