# Supplementary material for: Comparative Analysis of Salmon Cell Lines and Zebrafish Primary Cell Cultures Infection with the Fish Pathogen Piscirickettsia salmonis
Source: Microorganisms. 2021 Dec 6;9(12):2516. doi: 10.3390/microorganisms9122516 (PMC8706985; doi:10.3390/microorganisms9122516)
Supplement: Supplementary file 1 [file microorganisms-09-02516-s001.zip › Table S2.pdf]

S2 Table. Primers used for RT-qPCR in this study

Piscirickettsia salmonis						
**Gene name**	**Gene symbol**	**Forward sequence 5`→3`**	**Reverse sequence 3`→5`**	**Primer efficiency**	**Locus tag**	**Reference**
outer membrane protein	*ompA*	CCCCAGCTTTCACCATTAAA	AATTGGATAGTGCCCAGCAG	2.035	PSLF89_RS23070	Ortiz-Severin et al., 2020
flagellin	*fliC*	CACTGATCGCGCAAATCTAA	ATTGCGCTGCCTGTAATACC	1.989	PSLF89_RS23485	
fibronectin-binding protein	*cadF*	TGCAGTCATTTGTGCTGGAT	AAACGAAATGGTGCATAGCC	1.996	PSLF89_RS29915	
chemotaxis protein	*cheY*	GTTGGGCTCGCCTTATTAGA	AGCAGACACCACATCGGTTT	1.982	PSLF89_RS30125	
invasion associated protein	*iap/cwha*	GGTATGACGGACGCTTTGAT	ATAGGCTGCTAAGGCAAGCA	2.000	PSLF89_RS25340	
mammalian cell entry	*mce2B*	CGTCCGTTAGAATTGCTGGT	TGCTTGCCGAACTATCATCA	2.014	PSLF89_RS29945	
penicillin-binding protein 1A	*pbp1A*	CTAAGCGCACCCAAGAACTC	GACGGTGGTGACAACATCTG	1.986	PSLF89_RS33080	
tetraacyldisaccharide 4'-kinase	*lpxK*	CACGAGGTTATGGCGCTAAT	GGCACACCCGTTAGTTGTTT	2.030	PSLF89_RS25880	
glutamate-1-semialdehyde aminotransferase	*hemL*	TGGTGCACTCCTGATCTTTG	CAACAGGCATACCTCCACCT	2.014	PSLF89_RS35745	
ferric iron reductase	*fhuF*	TGGCCATCAGGTTCAATCTT	AACTGGCTTGGGTATTGACG	2.029	PSLF89_RS24790	
(p)ppGpp synthase/hydrolase	*relA*	TGCGTATTATGGTCGGTGAA	ACCATTTTCTTTTGGCGTTG	2.021	PSLF89_RS22790	
stationary phase specific sigma factor	*rpoS*	CGCGAGATTTGTCTCAATCA	GCCACAGGATCTGCCATATT	2.016	PSLF89_RS25070	
two component response transcriptional regulator	*mprA*	GAGAGCGAGCCTGTCAATCT	CAACCGTAGAGGGATTGCAT	2.008	PSLF89_RS27550	
bifunctional (p)ppGpp synthase/hydrolase	*spoT*	ATGCTGGACGCGGTATTATC	GACATTTCCACACGAACACG	1.984	PSLF89_RS28815	
stringent starvation protein A	*sspA*	TTTACGCTGGTTGATTGCAG	TGCATCACGAGAAAACAAGC	1.999	PSLF89_RS19855	
type IV secretion system protein	*icmE*	CGCTTAAGTGCACAGGATCA	TATCCCAATCACCCACCATT	2.031	PSLF89_RS30855	
type IV secretion system protein	*icmG*	CCAGGTTAAATTGTTGGTTGC	CAAACCCAGAGCTCGTGATA	1.988	PSLF89_RS30850	
type IV secretion system protein	*icmJ*	TGATGATCGCAATGGCTAAA	ACCAGAAACCCGACTCAATG	2.023	PSLF89_RS30800	
type IV secretion system protein	*icmB*	TGCCGATATTGGCTGATACA	GGGCTCGTTAGTGTTGGAAA	2.012	PSLF89_RS30790	
type IV secretion system protein	*icmG*	AAGGCCGATGTTGCATTATT	GCCTTTCGCTGACTTACTGC	2.007	PSLF89_RS24230	

type IV secretion system protein	*icmP*	TCGACGACCCATGATATGAA	TTTCTTCACCAACCCAAACC	1.990	PSLF89_RS24275	
type IV secretion system protein	*icmB*	GATATTGGGCCTTCAAGCAA	GCCCCTAACTGCGTATCAAA	1.988	PSLF89_RS24290	
type IV secretion system protein	*icmV*	CATTGATGACGGCAATTATAGG	CAAGCTGGCCGACTTTAATC	2.003	PSLF89_RS24320	
murine toxin	*ymt*	ATGTGCAATGGCTATGCTGA	ATCAGGCCAACTTTCCAATG	2.023	PSLF89_RS34605	
cell filamentation protein	*fic*	CAGCCTCGTATGATTGACCA	GGCCATTACCATCAGCAAAT	2.008	PSLF89_RS34625	
secreted effector protein	*pipB2*	GCGATGCCACACCTAAATCT	TTCTTGTGGCAAATGAGCAG	1.983	PSLF89_RS34855	
secreted effector protein	*pipB2*	TGGGTGGAATCTTGAAGGAG	TTTTTCGCAACAGTGCCTAA	1.998	PSLF89_RS34870	
secreted effector protein	*pipB2*	GCGATGCCACACCTAAATCT	TTCTTGTGGCAAATGAGCAG	2.000	PSLF89_RS35360	
endopeptidase	*pepO*	TGAAGACCGTGGATACACACA	GGGAAGAGGTTGAAGAGTCG	2.020	PSLF89_RS34880	
enterotoxin	*ospD3*	AGCGGCTTATATCCATGGTG	AATAGGTTTCTGCCGCTTGA	1.987	PSLF89_RS27860	
serine/threonine protein kinase	*pkn5*	TTTTGGCTGGAAGAAGGCTA	TGCTGCGATATCTTCGTTTG	1.979	PSLF89_RS28440	
DNA replication and repair family protein	*recF*	CGCCTTCAAGCCAATTGTGG	GCAAGCTTTTCACCTTGCCA	2.003	PSLF89_RS33770	
Transcription termination factor	*rho*	GCCAAGCGTTTGGTTGAACA	CTGAAGATGGTGCGACCGTA	2.098	PSLF89_RS32915	
Salmo salar						
**Gene name**	**Gene symbol**	**Forward sequence 5`→3`**	**Reverse sequence 3`→5`**	**Primer efficiency**	**Accession number**	**Reference**
interleukin 1, beta	*il1b*	GGAGAGGTTAAAGGGTGGCG	TCCTTGAACTCGGTTCCCAT	2.058	AY617117	Schiøtz et al., 2009
interleukin 6	*il6*	CAGCTTCTTCTTCAGCACGTTAA	CGTAGACACCTCACCCAGAAC	2.008	HF913655	Zante et al 2015
interleukin 8	*il8*	AGCGGCAGATTCAAACTCAC	GTTGTTGGCCAGCATCTTCT	1.984	BT046706	Reyes-Cerpa et al., 2012
interleukin 10	*il10*	GAACTCCGCACATCCTTCTC	CGTTGATGTCAAACGGTTTCT	1.993	EF165029	Reyes-Cerpa et al., 2012
interleukin 12	*il12*	CTGAATGAGGTGGACTGGTATG	ATCGTCCTGTTCCTCCG	2.001	BT049114	Hynes et al., 2011
interferon alpha	*ifnα*	CCTGCCATGAAACCTGAGAAGA	TTTCCTGATGAGCTCCCATGC	2.022	XM_014192435.1	Jørgensen et al., 2007

interferon gamma	*ifnγ*	TTCAGGAGACCCAGAAACACTAC	TAATGAACTCGGACAGAGCCTTC	2.016	AY795563	Jørgensen et al., 2007
tumor necrosis factor alpha	*tnfα*	CCAAACATTGGCTTGCACGA	AGCAGCAGGTCCTGAGAGTA	1.990		Pulgar et al., 2015
selenoprotein Pa	*selPa*	TGGCTGGAAAGAAGGATGAC	TGCTCTCATACGTGCAGTCC	2.003		Pulgar et al., 2015
interferon-induced guanylate binding protein 1,	*gbp1*	CGTCAATCAGCTGTCAGAGAACCA	TCGGAGGCATCCTTGTTCTGTTTG	2.005		Alvares et al., 20176
cathepsin D	*cstD*	GCCTGTCATCACATTCAACCT	CCACTCAGGCAGATGGTCTTA	1.993	U90321	Alvares et al., 20176
hepcidin	*hamp*	CATTGAAAATCGTGCATTGG	AAGGCCTTCATTCTCGGTTT	1.998		Pulgar et al., 2015
transferrin receptor	*trfr*	TTGTCGCAACCCCTATAACC	AAGACAGCCCACATCAGGTC	2.001		Pulgar et al., 2015
ferritin, lower subunit	*ferl*	TGTGCATGCATTTCCGTTAT	TTCAGTGCGAACCCATTACA	2.004		Pulgar et al., 2015
18S RNA	*18S*	TGTGCCGCTAGAGGTGAAATT	GCAAATGCTTTCGCTTTCG	1.985	AJ427629.1	Jørgensen et al., 2007
elongation factor 1 alpha	*eef1a*	CACCACCGGCCATCTGATCTACAA	TCAGCAGCCTCCTTCTCGAACTTC	2.000	AF321836	Jørgensen et al., 2007
Danio rerio						
**Gene name**	**Gene symbol**	**Forward sequence 5`→3`**	**Reverse sequence 3`→5`**	**Primer efficiency**	**Accession number**	**Reference**
interleukin 1, beta	*il1b*	QuantiTect Primer Assay Dr_il1b_1_SG		2.027	NM_212844,	Tandberg et al., 2016
interleukin 8	*il8*	QuantiTect Primer Assay Dr_il8_1_SG		2.004	XM_001342570,	Tandberg et al., 2016
interleukin 10	*il10*	QuantiTect Primer Assay Dr_il10_1_SG		1.999	NM_001020785	Tandberg et al., 2016
interleukin 12a	*il12*	QuantiTect Primer Assay Dr_il12a_1_SG		1.998	NM_00100710	Tandberg et al., 2016
interferon phi 1	*ifnΦ1*	QuantiTect Primer Assay Dr_ifnphi1_SG		1.981	NM_207640,	This study
interferon phi 2	*ifnΦ2*	QuantiTect Primer Assay Dr_ifnphi2_SG		2.026	NM_00111108	This study

interferon phi 3	*ifnΦ3(1)*	QuantiTect Primer Assay Dr_ifnphi3_1_SG		2.024	NM_001111083	This study
interferon phi 3	*ifnΦ3(2)*	QuantiTect Primer Assay Dr_ifnphi3_2_SG		2.032	NM_00111108	This study
interferon gamma	*ifnγ1-1*	QuantiTect Primer Assay Dr_ifng1-1_1_SG		1.992	NM_001020793	This study
interferon gamma	*ifnγ1-2*	QuantiTect Primer Assay Dr_ifng1-2_1_SG		2.040	NM_212864	Tandberg et al., 2016
tumor necrosis factor alpha	*tnfα*	QuantiTect Primer Assay Dr_tnfa_1_SG		1.997	NM_212859	Tandberg et al., 2017
iNOS (inducible nitric oxide synthetase)	*nos2a*	QuantiTect Primer Assay Dr_nos2a_1_SG		2.024	NM_001104937	This study
selenoprotein Pa	*sepp1a*	QuantiTect Primer Assay Dr_sepp1a_1_SG		1.951	NM_178297	This study
interferon-induced guanylate-binding protein 1, GBP1	*gbp1*	QuantiTect Primer Assay Dr_gbp1_1_SG		1.976	NM_001002343 XM_005171375, XM_005171376	This study
cathepsin D	*ctsd*	Dr_ctsd_1_SG QuantiTect Primer Assay		2.046	NM_131710, XM_005159140	Sieger et al., 2009
hepcidin	*hamp1*	QuantiTect Primer Assay Dr_hamp1_1_SG		1.993	NM_205583, XM_005170766 XM_005170767	This study
transferrin receptor	*tfr2*	QuantiTect Primer Assay Dr_tf2_1_SG		1.992	NM_001009916	This study
transferrin	*tfa1*	QuantiTect Primer Assay Dr_tfa1_1_SG		2.023	NM_001015057 XM_005168411 XM_005168412	This study
ferritin	*fth1a*	QuantiTect Primer Assay Dr_fth1a_1_SG		1.975	NM_131585, XM_005166370, XM_005166371	This study
interleukin 6	*il6*	TCAACTTCTCCAGCGTGATG	TCTTTCCCTCTTTTCCTCCTG	2.033	NM_001114318	Tandberg et al., 2016
myeloperoxidase	*mpx*	TGATGTTTGGTTAGGAGGTG	GAGCTGTTTTCTGTTTGGTG	2.010	NM_212779	Tandberg et al., 2016
macrophage expressed gene 1	*mpeg-1*	TACAGCACGGGTTCAAGTCCGT	ACTTGTGATGACATGGGTGCC G	1.990	NM_212737	Tandberg et al., 2016
18S rRNA	zgc:158463	GCCTGCGGCTTAATTTGACT	ACCACCCACAGAATCGAGAAA	1.948	NM_001098396	Tandberg et al., 2016

Eukaryotic translation elongation factor 1 alpha 1, like1	*eef1α111*	CTT CTC AGG CTG ACT GTG C	CCG CTA GCA TTA CCC TCC	2.035	NM_131263.1	Tandberg et al., 2016

Alvarez, C.A.; Gomez, F.A.; Mercado, L.; Ramirez, R.; Marshall, S.H. *Piscirickettsia salmonis* imbalances the innate immune response to succeed in a productive infection in a salmonid cell line model. PLoS One 2016, 11, 1.

Hynes NA, Furnes C, Fredriksen BN, Winther T, Bøgwald J, Larsen AN, Dalmo RA. Immune response of Atlantic salmon to recombinant flagellin. Vaccine 2011, 29, 7678.

Jørgensen SM, Hetland DL, Press CM, Grimholt U, Gjøen T. Effect of early infectious salmon anaemia virus (ISAV) infection on expression of MHC pathway genes and type I and II interferon in Atlantic salmon (*Salmo salar* L.) tissues. Fish Shellfish Immunol. 2007, 23, 57.

Ortiz-Severín, J.; Travisany, D.; Maass, A.; Verónica Cambiazo; Chávez, F.P. Global proteomic profiling of *Piscirickettsia salmonis* and salmon macrophage-like cells during intracellular infection. Microorganisms 2020, 8, 1845.

Pulgar, R.; Hödar, C.; Travisany, D.; Zuñiga, A.; Domínguez, C.; Maass, A.; González, M.; Cambiazo, V. Transcriptional response of Atlantic salmon families to *Piscirickettsia salmonis* infection highlights the relevance of the iron-deprivation defence system. BMC Genomics 2015, 16, 495.

Reyes-Cerpa S, Reyes-López FE, Toro-Ascuy D, Ibañez J, Maisey K, Sandino AM, Imarai M. IPNV modulation of pro and anti-inflammatory cytokine expression in Atlantic salmon might help the establishment of infection and persistence. Fish Shellfish Immunol. 201, 32, 291.

Schiøtz BL, Baekkevold ES, Poulsen LC, Mjaaland S, Gjøen T. Analysis of host- and strain-dependent cell death responses during infectious salmon anemia virus infection *in vitro*. Virol J. 2009, 6, 91.

Tandberg JI, Lagos LX, Langlete P, Berger E, Rishovd AL, Roos N, Varkey D, Paulsen IT, Winther-Larsen HC. Comparative analysis of membrane vesicles from three *Piscirickettsia salmonis* isolates reveals differences in vesicle characteristics. PLoS One 2016, 11, e0165099.

Tandberg J, Oliver C, Lagos L, Gaarder M, Yáñez AJ, Ropstad E, Winther-Larsen HC. Membrane vesicles from *Piscirickettsia salmonis* induce protective immunity and reduce development of salmonid rickettsial septicemia in an adult zebrafish model. Fish Shellfish Immunol. 2017, 67, 189.

Zante MD, Borchel A, Brunner RM, Goldammer T, Rebl A. Cloning and characterization of the proximal promoter region of rainbow trout (*Oncorhynchus mykiss*) interleukin-6 gene. Fish Shellfish Immunol. 2015, 43, 249.

Sieger, D., Stein, C., Neifer, D., van der Sar, A. M., & Leptin, M. The role of gamma interferon in innate immunity in the zebrafish embryo. Disease models & mechanisms.2009, 2(11-12), 571-581.
