# Supplementary material for: Comparative Analysis of Salmon Cell Lines and Zebrafish Primary Cell Cultures Infection with the Fish Pathogen Piscirickettsia salmonis
Source: Microorganisms. 2021 Dec 6;9(12):2516. doi: 10.3390/microorganisms9122516 (PMC8706985; doi:10.3390/microorganisms9122516)
Supplement: Supplementary file 1 [file microorganisms-09-02516-s001.zip › TableS1.pdf]

Table S1. Quality of RNA samples measured by absorbance 260/280 ratio.

Sample	Absorbance (ratio 260/280)
Control (mock-infected)	
ASK 6 dpi (1)	1.99
ASK 6 dpi (2)	2.15
ASK 6 dpi (3)	2.08
ASK 12 dpi (1)	2.43
ASK 12 dpi (2)	2.30
ASK 12 dpi (3)	2.17
SHK-1 6 dpi (1)	2.19
SHK-1 6 dpi (2)	2.10
SHK-1 6 dpi (3)	2.04
SHK-1 12 dpi (1)	2.18
SHK-1 12 dpi (2)	2.08
SHK-1 12 dpi (3)	2.13
ZKPCC 1 dpi (1)	2.24
ZKPCC 1 dpi (2)	2.13
ZKPCC 1 dpi (3)	2.19
ZKPCC 5 dpi (1)	2.03
ZKPCC 5 dpi (2)	2.03
ZKPCC 5 dpi (3)	2.17
Infection (*P. salmonis* LF-89)	
ASK 6 dpi (1)	2.21
ASK 6 dpi (2)	2.13
ASK 6 dpi (3)	2.17
ASK 12 dpi (1)	2.19
ASK 12 dpi (2)	2.22
ASK 12 dpi (3)	2.17
SHK-1 6 dpi (1)	1.98
SHK-1 6 dpi (2)	2.01
SHK-1 6 dpi (3)	2.15
SHK-1 12 dpi (1)	2.18
SHK-1 12 dpi (2)	2.16
SHK-1 12 dpi (3)	2.16
ZKPCC 1 dpi (1)	2.25
ZKPCC 1 dpi (2)	2.18
ZKPCC 1 dpi (3)	2.17
ZKPCC 5 dpi (1)	2.14
ZKPCC 5 dpi (2)	2.03
ZKPCC 5 dpi (3)	2.19
P. salmonis LF-89 in Austral-SRS medium	
P. salmonis (1)	2.09
P. salmonis (2)	2.07
P. salmonis (3)	2.19
