# Supplementary figures and images for: Comparative Analysis of Salmon Cell Lines and Zebrafish Primary Cell Cultures Infection with the Fish Pathogen Piscirickettsia salmonis
Source: Microorganisms. 2021 Dec 6;9(12):2516. doi: 10.3390/microorganisms9122516 (PMC8706985; doi:10.3390/microorganisms9122516)

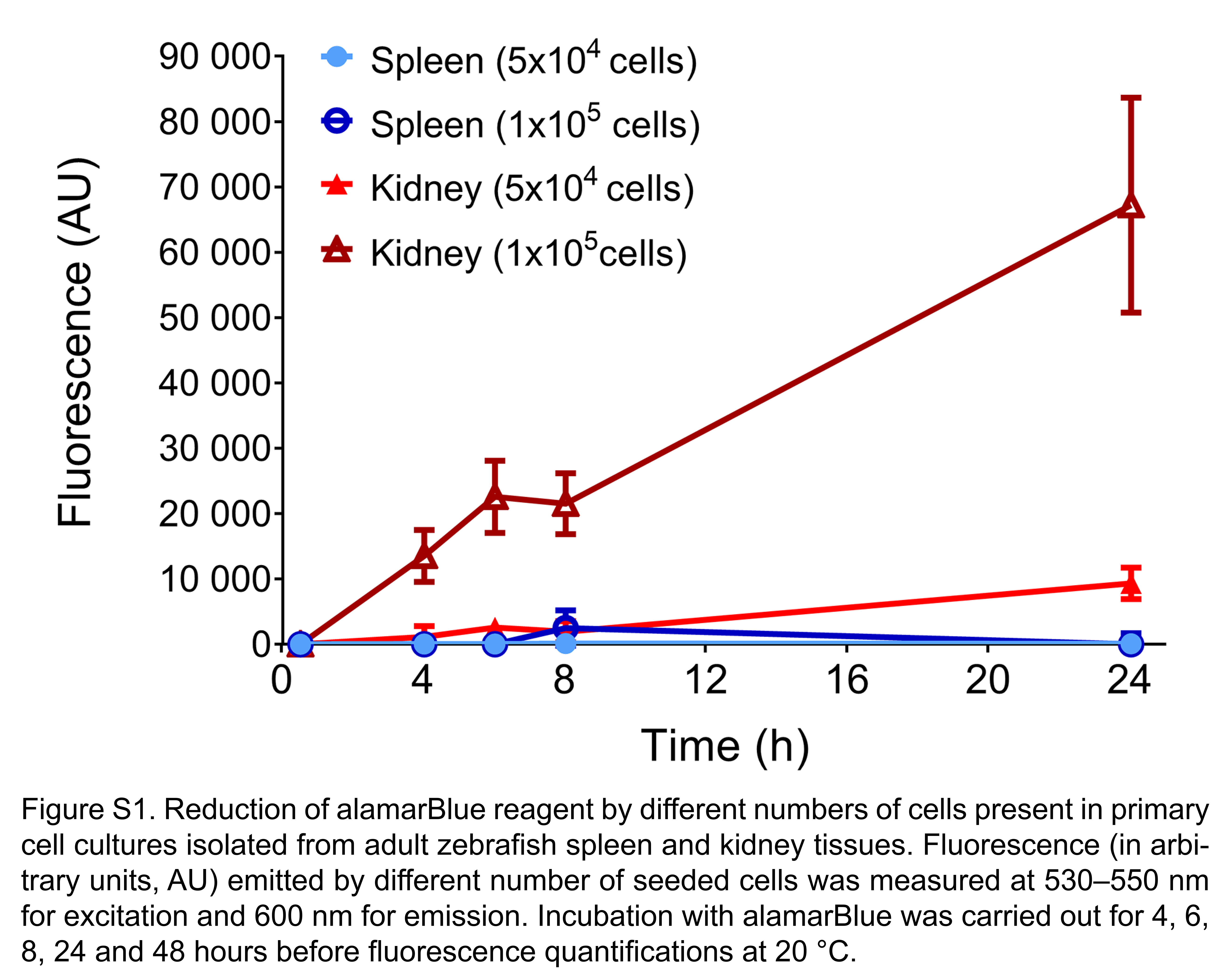

Supplement: Supplementary file 1 [file microorganisms-09-02516-s001.zip › FigureS1.tif]

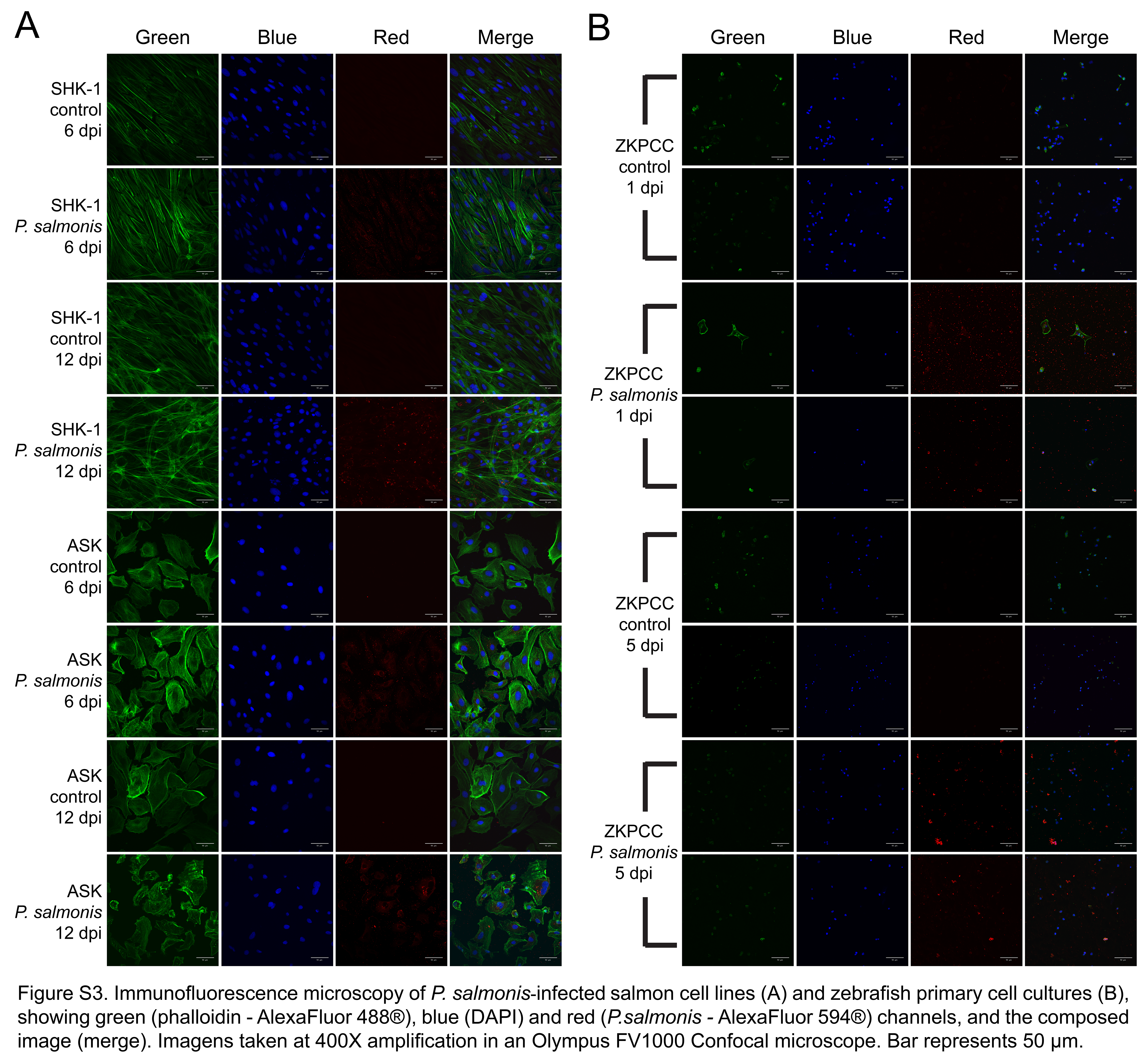

Supplement: Supplementary file 1 [file microorganisms-09-02516-s001.zip › FigureS3.tif]
